# A Handbook of Ophthalmic Science and Practice

**Published:** 1904-06

**Authors:** 


A HANDBOOK OF OPHTHALMIC SCIENCE AND PRACTICE. Third Edition. Revised and enlarged. By HENRY E. JULER, F.R.C.S., Ophthalmic Surgeon to St. Mary's Hospital; Consulting Surgeon to the Royal Westminster Ophthalmic Hospital; Consulting Ophthalmic Surgeon to the London Lock Hospitals. Octavo, 733 pages, with 190 illustrations and 25 full-page plates in colors and black. Lea Brothers & Company, Philadelphia and New York. 1904. (Cloth, $5.25 net.)

It is now twenty years since the first edition of Juler's Handbook was published. From the outset it has been recognised by ophthalmologists and teachers as one of the most complete and trustworthy works on the eye. During all of this time the English medical schools have made it their leading textbook. It covers the whole range of ophthalmology, including a full exposition of the anatomy and physiology of the eye. Mr. Juler has the faculty of expressing himself concisely and at the same time clearly, and his skill in compressing a large amount of information into a small space is certainly quite unusual.

The present edition has been brought fully up to date, and includes an intelligible reference to almost every known disease of the eye. It is most excellently illustrated, both in black and colors, and is in every way well gotten up. To those who desire a reliable and complete work in this department of medical science, this one is to be commended. Indeed, there are few books on the subject that contain so much that is of practical use to the student and practitioner. A. A. H.

